# Role of neuropsychological assessment in the differential diagnosis of Alzheimer’s disease and vascular dementia

**DOI:** 10.1590/S1980-57642009DN30300007

**Published:** 2009

**Authors:** Érica Maria Lima Pimentel

**Affiliations:** Universidade Federal do Ceará (UFC) e Pós-graduada em Neuropsicologia pela Faculdade Christus, Fortaleza, CE, Brazil.

**Keywords:** Alzheimer's disease, vascular dementia, neuropsychology, diagnosis

## Abstract

The prevalence of dementia increases significantly from the age of 65 years, doubling every five years thereafter. Alzheimer’s disease (AD) and vascular dementia (VaD) constitute the two main dementia types. Differentiating them encompasses anamnesis, neurological examination, laboratory and neuroimaging exams and neuropsychological assessment. Neuropsychological assessment produces different findings for each dementia type, and reveals those areas most impaired as well as those most preserved. The aim of the present article was to describe the role of neuropsychology in diagnosing dementia and achieving a differential diagnosis between AD and VaD. A general overview follows of the most widely known instruments used to assess cognitive function in dementia, and the cognitive changes seen in AD and VaD. The conclusion drawn was that there is significant overlap in cognitive changes between both these dementia types, while each type has its own specific characteristics which are identifiable and quantifiable on neuropsychological assessments and provide the basis for reaching a differential diagnosis.

## Abstract (pt)

A prevalência de demência aumenta de maneira expressiva a partir dos 65 anos, dobrando a cada cinco anos. Doença de Alzheimer (DA) e demência vascular (DVa) são os seus dois principais tipos. O diagnóstico diferencial entre DA e DVa engloba anamnese adequada, exame neurológico, exames laboratoriais e de neuroimagem e avaliação neuropsicológica. Dentro da avaliação neuropsicológica, é possível observar as diferenças nos achados para cada demência, as áreas mais comprometidas e aquelas que ainda estão preservadas. O presente artigo tem como objetivo descrever a contribuição da neuropsicologia para o diagnóstico das demências e para o diagnóstico diferencial entre DA e DVa. É apresentada uma visão geral dos instrumentos mais conhecidos e utilizados para avaliar a função cognitiva na demência e as alterações cognitivas encontradas na DA e na DVa. Conclui-se que existe importante sobreposição das alterações cognitivas em ambas, contudo cada uma possui características particulares que podem ser identificadas e quantificadas pela avaliação neuropsicológica e que contribui para o diagnóstico diferencial.

The ageing of the population has given rise to a host of medical conditions such as different types of dementia. Alzheimer’s disease (AD) and vascular dementia (VaD) are the most common causes of dementia and reaching a differential diagnosis is key to the treatment and management of these patients. The diagnosis is reached thorough a combination of anamnesis, neurological examination, laboratorial and neuroimaging exams and neuropsychological assessment.^12^

Neuropsychology is a recent discipline formed by the merging of Neurology and Psychology. Neuropsychological assessment currently concentrates on ascertaining the extent and impact of cognitive and behavioral repercussions, as well as psychosocial adaptation, caused by cerebral lesions or dysfunctions.^21^ Neuropsychological batteries are based on a combination of instruments which assess cognitive and behavioral functions. Behavior can be defined in terms of three functional systems: cognition, emotion and executive functions.^35^ This assessment is important to support the differential diagnosis and prognosis, enabling orientation for treatment and planning of rehabilitation.

The present article explores the role of Neuropsychology in the differential diagnosis of the most common causes of dementia. To this end, the general concept of dementia and its classification shall first be addressed, while AD and VaD will subsequently be explored, providing an overview on their respective diagnosis, course and the instruments most widely used to assess them, along with the most significant findings of each in terms of those functions most impaired.

## Study on dementia

### Definition and classification of dementia

Dementia can be defined as a clinical syndrome characterized by global decline of superior cortical functions which compromise social and occupational functioning of the individual.^42^ The Brazilian Academy of Neurology (ABN)^44^ recommends that the clinical diagnosis of dementia be based on the criteria of the 4th edition of the Diagnostic and Statistical Manual of Mental Disorders by the American Psychiatric Association (DSM-IV).^63^ In order to be diagnosed with dementia, the individual must present with prior decline in functioning as a result of memory impairment, and impairment of at least one cognitive function: language, agnosia, praxis, executive function or spatial function. Also, these deficits must not occur exclusively during acute confusional syndrome or *delirium* pictures.^17,43^ However, it should be emphasized that all types of dementia stem from Central Nervous System compromise (CNS).

Dementia is more prevalent in the elderly population. Prevalence of the disease doubles every five years beyond the age of sixty-five.^21^ A study in a Brazilian population conducted by Herrera et al. (2002)^30^ reported a prevalence ranging from 1.6% in elderly aged between 65 and 69 years, to 38.9%, among individuals aged over 84 years.

Dementia can be classified into primary and secondary.^21,42^ The former are known as neurodegenerative since they constitute dementia as a clinical manifestation. AD belongs to this first group and is the leading cause of dementia, accounting for fifty to seventy percent of all dementia cases. Dementia with Lewy bodies and frontal-temporal lobe degeneration are also part of this group. The primary dementias also include those in which dementia is set to become the main clinical manifestation. Such examples include Parkinson’s disease, Huntington’s disease and progressive supranuclear palsy.

Secondary forms comprise non-degenerative dementias, since they stem from an array of different clinical conditions. The most important type in this group is VaD which represents approximately twenty percent of all dementias, making it the second leading cause of the syndrome. Other secondary dementias include infectious processes (neurosyphilis, HIV-associated dementia), metabolic (hyperthyroidism, Vitamin B12 deficiency) and those which alter brain structure (tumor, swelling, hydrocephalus).

The differential diagnosis must identify the potentially reversible pictures of a variety of etiologies such as metabolic alterations, infections, metabolic deficiencies, intoxications etc.^23^ A rigorous clinical examination including an in-depth anamnesis, neurological exam and neuropsychological assessment, besides complementary investigation comprising laboratorial and neuroimaging exams all contribute to a more accurate differential diagnosis.^12^

The Brazilian Association of Neurology recommends the following laboratory exams: complete hemogram, sera levels of urea, creatine, free thyroxin (T4), thyroid stimulating hormone (TSH), albumin, hepatic enzymes (TGO, TGP, Gama-GT), Vitamin B12 and calcium, serological reactions for syphilis and, in patients aged younger than 60 years, serology for HIV. Cerebral Spinal Fluid exam (CSF) may be useful for identifying the specific causes of dementia.

Neuroimaging exams, such as computerized tomography and/or magnetic resonance are used to rule out other possible diagnoses and co-morbidities. Perfusion scintigraphy (SPECT) and electroencephalogram (EEG) are recommended as auxiliary methods.^44^

Differential diagnosis with other psychiatric disorders is also important.^44^ The *Geriatric Depression Scale* (GDS)^60^ or the Cornell^3^ scale as a second option, are recommended to screen for depression, whereas the *Confusion Assessment Method* (CAM) scale^18^ is recommended for diagnosing *delirium*.

Cognitive assessment typically starts with simple tests or screening instruments such as the Mini-Mental-State-Exam (MMSE).^10,20^ In the event of poor performance on the MMSE, it is essential to carry out a more in-depth complementary assessment using the Neuropsychological Assessment by applying tests which assess multiple cognitive domains including attention, memory, language, perception, executive functions, visuoconstructive function and visuospatial function. Other available batteries include the 3^rd^ edition of the Wechsler Adult Intelligence Scale (WAIS-III)^41^, the *Cambridge Cognitive Examination* (CAMCOG),^9,51^ *Consortium to Establish a Registry of Alzheimer’s Disease* (CERAD) Test battery,^7,40^ and the *Alzheimer’s Disease Assessment Scale-Cognitive Subscale* (ADAS-cog).^56^ All of these tests cover the domains outlined above. Brief test may also be applied which are quick and easy to apply, such as the clock-drawing test, memory tests on figures or word lists, and verbal fluency tests.^35^

Functional assessment aims to correlate cognitive deficits with their impact on activities of daily living. This may be performed during anamnesis and also while applying questionnaires, such as the Pfeffer Functional Activities Questionnaire46 or Lawton and Brody’s Questionnaire on Activities of Daily Living.^42^

### Alzheimer’s disease (AD)

Alzheimer’s disease is the most commonly occurring and widely-studied form of dementia. AD presents in individuals from the age of 65 years, although may occasionally occur earlier in patients as young as 45 years old.^21^ The Picture evolves insidiously, a fundamental characteristic of AD which Engel et AL. (2003)17 proposed to differentiate the disease.

In order to diagnose Alzheimer’s disease, the Brazilian Academy of Neurology recommends the adoption of the criteria of the *National Institute for Communicative Disorders and Stroke-Alzheimer’s Disease* and *Related Disorders Association* (NINCDS-ADRDA) by McKhan et al. (1984).^38^

This encompasses the following criteria:

- Dementia determined by clinical exams and documented by the Mini Mental State Exam, the Blessed dementia scale, or a similar assessment, and confirmed by neuropsychological tests.
- Deficits in two or more cognitive areas;
- Progressive decline in memory and other cognitive functions;
- Absence of delirium;
- Onset between 40 and 90 years, predominantly after the age of 65 years; and
- Absence of systemic diseases or other cerebral diseases which may independently cause progressive decline in memory and cognition.

It is noteworthy that these deficits have to affect the performance in activities of daily living. The neuropsychological assessment includes specific tests designed to evaluate each function which will be examined in more detail later in this text.

Other behavioral changes take place during the course of the disease, such as mood disorders, sexual changes, changes in appetite, delirium and hallucinations, apathy and indifference, disinhibition, disturbances in psychomotor activity, and sleep disturbances.^4^ The most commonly found symptoms in Alzheimer’s disease are memory impairment, loss of topographic orientation, personality changes^33^ and language disturbances.^33,42^ The alterations in cognitive functions in AD will be examined in more detail in item three of this article.

AD is characterized by cortical atrophy which chiefly affects hippocampal formation and associative cortical areas.^13,53^ This distribution of the pathological process coupled with the relative sparing of the primary cortices allows the clinical picture of AD to be characterized by cognitive and behavioral alterations, with preserving of motor and sensory functioning until more advanced stages of the disease.^12^ Microscopy exams reveal the presence of senile plaques, neurofibrillary tangles, neuronal loss and characteristic histological changes. These anatomopathological findings are essential for the definitive diagnosis of this disease.

Although these pathological alterations permeate the entire cerebral cortex, the temporal, frontal and parietal lobes are the most severely affected regions.^33^

### Vascular dementia (VaD)

VaD is the second leading cause of dementia in the elderly, accounting for around twenty per cent of all cases.^21^ The term VaD covers a multitude of dementia syndromes secondary to cerebrovascular compromise.^42^ The disease can be caused by multiple thromboembolic lesions, small vessel lesions, single lesions in key locations (such as the thalamus or left angular girus), lacunar infarcts, chronic alterations in cerebral circulation, extensive white matter lesions (Binswanger disease), amyloid angiopathy and intercerebral hemorrhagic lesions.) Since VaD is secondary to cerebrovascular compromise, it is possible to perform primary and secondary prevention.^55^

Prevalence of VaD varies considerably among different populations.^2^ A Canadian study 11 reported that 19% of dementia cases were VaD. The prevalence found in a Rotterdam study was 14%45 while in the Japanese population was 56%.^57^ An epidemiological study conducted in Brazil by Herrera et al. (2002)^30^ found a 9.3% prevalence of VaD.

The main instruments used to classify VaD are: DSM-IV,^63^ the *State of California Alzheimer’s Disease Diagnostic and Treatment Centers* (ADDTC)^62^ and the *National Institute of Neurological Disorders and Stroke and Association Internationale pour La Recherche et Le Enseignemant em Neurosciences* (NINDS-AIREN).^50^ We shall concentrate only on this last scale since it contains the latest and most specific diagnostic criteria.^56^

The diagnostic criteria for vascular dementia according to the NINDS-AIREN, address three core aspects: demential syndrome, cerebrovascular disease, and the time relationship between the two. The criterion defining the presence of dementia is memory compromise in at least two domains which is sufficient to interfere in activities of daily living. Cerebrovascular Disease (CVD) is determined by focal neurological signs of CVD on the exam, in addition to positive evidence of CVD on tomography or resonance imaging. Finally, the NINDS-AIREN deems the onset of dementia as within three months of a stroke (CVA) with abrupt deterioration of cognitive functions and a course which fluctuates in a step-wise fashion.

In order to reach an accurate diagnosis, screening must be carried out which evaluates risk factors, manifestation of a cerebrovascular episode and the occurrence of the dementia picture^5^ Engel et al. (2003)^17^ consider risk factors of dementia to be Systemic Arterial Hypertension (SAH), smoking, diabetes, alcoholism, heart disease, atherosclerosis, dyslipidemia and obesity. Other risk factors associated to VaD are black race, low schooling and male gender.^55^ Identifying these risk factors, as well as diagnosing early VaD, allows preventive strategies to be devised which may improve the evolution of the patient or prevent the onset of the disease.

The classic clinical picture of VaD is characterized by abrupt onset, secondary to a stroke (CVA) or a transient ischemic attack (TIA), exhibiting stability, improvement or progressive worsening, frequently of a fluctuating step-wise nature.^42^ The disease is commonly associated with focal neurological signs, such as hemiparesis, ataxia, hemianopsia, aphasia and hemineglect.^17^ These factors are very important to enable a differential diagnosis to be reached and require the use of Brain CT and magnetic resonance exams which generally reveal several cerebral infarcts. The Hachinski scale^28,29^ can be employed to complement the diagnosis. Scores greater than or equal to seven indicate a higher likelihood of vascular dementia while scores less than five suggest degenerative dementias such as AD.

## Most frequently used neuropsychological batteries and tests in dementia 

The neuropsychological tests outlined below were identified through a systemic review of the literature held on the Pubmed, Medline, and Lilacs scientific databases, as well as textbooks and test manuals. The functions altered in AD and VaD, respectively, are analysed and correlated.

The MMSE^6,10,20^ comprises questions grouped into seven categories: temporal-spatial orientation, retention or registering of data, attention and calculation, memory, language and visuoconstructive ability. The MMSE is a simple test which is quick to apply (duration of only five to ten minutes). Scores range from zero to thirty points where scores less than 24 points are suggestive of dementia (or *delirium*). The score obtained is strongly influenced by schooling and adjusting cut-off by educational level is recommended.^8,42^

Brucki et al., (2003)^10^ reported mean scores on the MMSE for the Brazilian population according to level of schooling. These authors observed a mean score of 19.51 points among illiterate individuals (standard deviation (SD) 2.84); a mean of 24.76 (SD 2.96) in individuals with schooling of four years; mean 26.15 (SD 2.35) in those with five to eight years of schooling; mean 27.74 (SD 1.81) in subjects with schooling of nine to eleven years, and mean score 28.27 (SD 2.01) in individuals with twelve or more years of education.

Other tests may also be used for screening. Azambuja (2007)^6^ recommended the use of the Brief Assessment of Cognitive Functions,^24^ which tests cognitive functions such as temporal-spatial orientation, attention and memory, comprehension, naming, repetition, reinforcement, mental calculation, reasoning and judgment, praxias, written order, verbal fluidity, visual and written decoding.

The clock drawing test can also serve as a screening test given its quick application. This test provides a more accurate assessment of the visuospatial function and executive function,^6,43^ but is also influenced by other cognitive functions. The test reveals the pattern of frontal and temporal-parietal functioning.^43^

The neuropsychological batteries and tests below are instruments which comprise the first assessment applied by the physician. These tests should be applied by specialized and qualified professionals who should exercise common sense when choosing each test and take into account factors which may influence the outcome such as visual or hearing impairments, level of schooling, differences in customs and cultures, or motor difficulties.

Memory (fixation, evocation and knowledge), language (naming and verbal fluency), praxis (copy of geometric drawings) and executive function (trail-making test) as sessments are incorporated into the CERAD battery, used to diagnose probable AD.^7^

The Mattis Dementia Rating Scale (DRS)^37^ contains 36 items under five subscales: attention, initiative/perseverance, construction, conception and memory. The scale is easy and quick to apply, taking between 30 and 40 minutes in patients with dementia.^36^ The Mattis DRS presents several advantages over other batteries47: it yields more detailed information on those cognitive functions which are preservcd^19,39^ because the assessment tests numerous cognitive areas in more depth and offers higher sensitivity for more severe forms of AD.^52^

A Brazilian study (Porto et al., 2003)^47^ concluded that the Brazilian version of the Mattis Dementia Rating Scale showed good diagnostic accuracy n discriminating between patients with mild AD and controls. The effects of schooling were found to be greater than those of age in the population studied.

The WAIS III41 is used in adults aged between sixteen and 89 years old. It contains fourteen subtests and four factorial indices (verbal comprehension, perceptual organization, working memory and processing speed). Besides being used to measure the intellectual coefficient, it can provide an analysis of each subtest and factorial index. The ADAS-cog is employed to assess AD and covers the most compromised domains in this disease such as attention, orientation, memory, visuospatial abilities and executive functions.^56^ The brief cognitive screening battery^58^ is a quick-to-apply mini-battery which assesses five aspects of memory: incidental memory, immediate memory, learning, delayed memory and recognition. The verbal fluency and clock drawing tests are also applied as part of this battery.

Although these batteries assess a broad range of functions, more specific tests aimed at investigating particular functions need to be used.

Loss of memory is an early symptom of AD and other dementia types. Fuentes et al. (2008)^21^ and Nitrini et al. (2005)^43^ suggested that delayed recall tests,^59^ the Rey Auditory-Verbal Learning test,^16,48^ *Fuld Object Memory Evaluation* (FOME)^22^ and logic memory test I and II of the Wechsler Memory Scale – WMS,^35^ are highly effective for assessing this function. The CAMCOG, CERAD and Mattis-DRS contain subtests which assess memory.

In order to assess attention, the same authors recommended the use of the Direct and Reverse digit tests (WAIS-III) which assess verbal attention, working memory and immediate memory.^21,43^ while the trail-making test^35,43^ is useful for evaluating selective attention, speed of perceptual processing and mental flexibility. The random letter test^35,43^ assesses awareness whereas the Stroop test^35,43^ is indicated for assessing divided attention. For assessing working memory, the serial subtraction of sevens from one hundred, and backwards spelling of common words test from the MMSE subitem are recommended.

Language is also found to be impaired in both AD and VaD. Language can be assessed using batteries devised to assess aphasia, such as the Boston Diagnostic Aphasia battery,^25^ *Westem aphasia Battery*,^32^ *Token Test*^15^ and the Boston Naming test.^31^ It should be noted that these techniques have yet to be standardized in the Portuguese language.

The Brazilian Academy of Neurology indicates, as a practical option, the use of the Boston Naming Test (15 items of the CERAD), the naming of real objects test from the ADAS-Cog or eight figure naming from the Brief Neuropsychological Assessment Battery (NEUROPSI)^1^ for assessing language in AD patients.

According to Fuentes et al., (2008)^21^ the verbal fluency test for semantic (animals) and phonological (FAS) categories, verbal abstraction, interpretation of proverbs and the Wisconsin Card Sorting test (WCST)^14^ are among the most commonly used tests for assessing executive functions, as is the clock drawing test. The same authors describe the use of free drawing, copying (Rey’s complex figure,^21,61^ Necker’s cube,^18,21,32^ pentagons of the MMSE and cube construction (WAIS III) for assessing visuospatial and constructive praxia functions. Although standardized tests of visuospatial functions are not available for the Brazilian population, Nitrini et al. (2005)^43^ recommends the use of descriptions of themed figures (e.g. the cookie theft test from the Boston Diagnostic Aphasia battery) or the overlapping figures perception test.^34^ The same neuropsychological tests employed to assess AD also appear to be applicable for VaD, with differences in quantitative and qualitative findings between the two diseases.

## Discussion on cognitive function alterations in AD and VaD

The evolution of Alzheimer’s disease may be divided into three stages. Changes in cognitive functions shall be analyzed for each stage of the disease course. Fuentes et al. (2008)^21^ defined loss of memory for recent facts, along with sparing of memory for remote facts as characteristics of the first stage of AD. The patient has problems recalling names and information, does not remember where they left personal items and may have problems handling money or paying bills. In this initial stage, these difficulties gradually evolve into impairments in other cognitive functions such as calculation, powers of judgment, visuospatial skills and abstract reasoning.^23^

Neuropsychological tests indicate compromise in semantic memory and reveal difficulties in verbal fluency tasks which also reflect problems in executive functions. It should be emphasized that the ability to retrieve information is influenced little by cueing. The chart below lists the main cognitive alterations along with their anatomic correlates, and main tests for assessing each function.

In the second stage, virtually all cognitive functioning shows evidence of compromise. Apraxia may occur^23^ and languages alterations may be evident,^54^ particularly anomia, aphasia, acalculia and agnosia. Comprehension also becomes altered, and disturbances in planning and logical reasoning may be observed. Both recent and past memory becomes severely compromised and impairment in Visuo-spatial abilities occur.^21^ The patient presents confusion, restlessness, loss of interest in habitual activities and gets lost easily, even within their own home.^26^

In the terminal stage, all cognitive functions are seriously affected. The patient presents echolalia, palilalia or mutism and there are alterations in balance, gait and muscle strength.^21,42^ Changes in sleep-wake states may also take place, along with behavioral alterations such as irritability and aggressiveness; psychotic symptoms; inability to walk, speak or perform self care.^23^ The patient may present muscular rigidity, urinary incontinence and clinical complications related to motor problems, such as difficulties in swallowing, aspiration pneumonia, or urinary infection with sepsis. These complications are generally those that lead to death.

Psychotic symptoms and agitation are more often seen in elderly patients, while disturbances in language and visual recognition are more present in pre-senile age groups.^42^

Vascular dementia can affect cortical and/or subcortical areas, where primary symptoms are deficits in executive functions.^49^ According to André (1998),^5^ patients who present VaD exhibit greater impairment in executive functions; may present psychiatric manifestations such as agitation and anxiety at any time during the course of the disease as well as sleep alterations and depression in the initial phase, and delirium in the final phase. According to the study by Groves et al. (2000),^27^ patients with VaD showed higher rates of depression and functional compromise, as well as low cognitive impairment compared to AD patients.

Neuropsychological findings reveal that VaD patients exhibit less impairment to episodic memory than AD patients but present a greater degree of impairment in attention, executive and motor functions.^62,63^ Compromise in phonemic verbal fluency has also been observed in VaD, while individuals with AD show deficits in semantic fluency.^21^

A study carried out by André (1998)^5^ also revealed differences in cognitive impairment between AD and VaD. According to the author, VaD patients present more severe anosognosia, greater emotional lability and worse impairment on tests of planning, sequencing and switching attention. Based on these findings, the author concluded that patients with VaD suffer from frontal and sub-frontal dysfunction.

## Conclusion

Dementia is an increasingly prevalent syndrome in everyday life. The leading form of dementia manifests as Alzheimer’s disease and may represent seventy percent of cases, followed by vascular dementia which accounts for twenty percent of dementias. There is substantial overlap of cognitive alterations among AD and VaD, although each dementia type has distinct distinguishing characteristics.

Neuropsychological assessment allows cognitive deficits to be identified and quantified and can track their evolution. These tests help reach a differential diagnosis and guide treatment and rehabilitation. Numeric data, scores and indices (quantitative data) obtained through neuropsychological assessment, together with data obtained from observation of behavior, strategies employed, reactions and expressions of the patient on tests (qualitative data) are fundamental and complementary toward achieving a more accurate assessment.^35^ Such assessments must be performed by trained professionals who exercise prudence in choosing the most suitable neuropsychological test and battery. Factors which may influence test outcomes should be allowed for such as: schooling, age group, drugs use, visual and hearing deficits, differences in customs and cultures, or motor difficulty.

## Figures and Tables

**Table 1:** Hachinski Ischemic Score.

Abrupt Onset	2
Step-wise deterioration	1
Fluctuating course	2
Nocturnal confusion	1
Relative preservation of personality	1
Depression	1
Somatic Complaints	1
Emotional Incontinence	1
History of arterial hypertension	1
History of Stroke (CVA)	2
Evidence of associated atherosclerosis	1
Focal neurologic symptoms	2
Focal neurologic signs	2

**Table 2:** Definition of the main cognitive functions altered in the initial phase of AD, neuroanatomical correlates, and most commonly used tests.

Episodic memory	Memory for personal experiences in a given context in time and space
Test	Logic Memory (WMS-III)
Neuroanatomy	→ Hippocampal formation
Semantic memory	Knowledge of public events, vocabulary and association of concepts
Test	Vocabulary (WAIS-III)
Neuroanatomy	→ Anterior temporal lobe and spread throughout neocortex
Executive functions	Coordination of multiple cognitive processes
Test	Wisconsin card sorting test
Neuroanatomy	→ Pre-frontal cortex
Working memory	Ability to manipulate data from short-term memory
Test	Reverse digits (WAIS-III)
Neuroanatomy	→ Pre-frontal cortex

Source: Fuentes et al., 2008.
